# Point-of-care ultrasound for evaluating intra-abdominal calcification in the pediatric emergency department: case series and review of literature

**DOI:** 10.1186/s13089-020-00199-y

**Published:** 2020-12-03

**Authors:** Vigil James, John Samuel, Chor Yek Kee, Gene Yong-Kwang Ong

**Affiliations:** 1 Children’s Emergency, C/O KK Women’s and Children’s Hospital PTE. LTD, 100 Bukit Timah Road, Singapore, 229899 Singapore; 2 Department of Radiodiagnosis, Christian Fellowship Hospital, Oddanchatram, Tamilnadu 624619 India; 3 Paediatric Intensive Care Unit, Department of Pediatrics, Sarawak General Hospital, Kuching, Malaysia

**Keywords:** Ultrasound, Intra-abdominal, Calcification, Pediatric emergency, Point-of-care ultrasound, POCUS

## Abstract

**Background:**

The presence of intra-abdominal calcification in the pediatric population can be due to a wide range of conditions. Calcification in the abdomen can be seen in normal or abnormal anatomical structures. In some patients, abnormal calcification points towards the pathology; whereas in others, calcification itself is the pathology. After a thorough history and clinical examination, point-of-care ultrasound (POCUS) would complement the assessment of acute abdominal pain, based on the list of differentials generated as per the abdominal region. The main objective of this article is to review commonly encountered causes of intra-abdominal calcifications in the pediatric population and help in clinical decision-making in a Pediatric Emergency Department.

**Case presentation:**

We describe a series of pediatric patients who presented to the Pediatric Emergency Department with acute abdominal pain, in whom point-of-care ultrasound helped expedite the diagnosis by identifying varying types of calcification and associated sonological findings. For children who present to the Pediatric Emergency Department with significant abdominal pain, a rapid distinction between emergencies and non-emergencies is vital to decrease morbidity and mortality.

**Conclusions:**

In a child presenting to the Pediatric Emergency Department with abdominal pain, POCUS and the findings of calcifications can narrow or expand the differential diagnosis when integrated with history and physical exam, to a specific anatomic structure. Integrating these findings with additional sonological findings of an underlying pathology might raise sufficient concerns in the emergency physicians to warrant further investigations for the patient in the form of a formal radiological ultrasound and assist in the patient's early disposition. The use of POCUS might also help to categorize the type of calcification to one of the four main categories of intra-abdominal calcifications, namely concretions, conduit wall calcification, cyst wall calcification, and solid mass-type calcification. POCUS used thoughtfully can give a diagnosis and expand differential diagnosis, reduce cognitive bias, and reduce physician mental load. By integrating the use of POCUS with the history and clinical findings, it will be possible to expedite the management in children who present to the Pediatric Emergency Department with acute abdominal pain.

## Background

The presence of intra-abdominal calcification in the pediatric population can be due to a wide range of conditions. Calcification in the abdomen can be seen in normal or abnormal anatomical structures. In some patients, abnormal calcification points towards the pathology; whereas in others, calcification itself is the pathology [1]. The presence of intra-abdominal calcification may be diagnostic in some patients, and in some patients, it might raise enough concerns to warrant further investigations in the patient.

Intraabdominal calcifications in children can occur due to a broad spectrum of conditions ranging from benign causes to adverse conditions like malignancy. These can be broadly divided into concretions, conduit calcifications, cystic calcifications, and solid mass calcifications [1]. Concretions are discrete precipitates in an organ or vessel like renal, ureteric, vesical, and gall bladder calculi. Conduit calcifications are calcifications seen in the walls of any fluid-filled hollow tubular structures like pancreatic duct calcifications and vascular wall calcifications [1]. The cystic calcifications arise in the wall of a cyst or pseudocyst. Solid mass calcification shows extensive, but variable calcification with a dense center and irregular margins like mesenteric lymph nodes, adrenals, and metastasis [1].

Intra-abdominal calcification is detected on the ultrasound by the presence of a hyperechoic area in the abdominal organs. Calcium obstructs ultrasound more than any natural substance and results in the formation of an area of sonological opacity (signal void) called the posterior acoustic shadowing [2]. Once the intra-abdominal calcification is detected on ultrasound, the addition of accessory sonographic features of specific pathologies will help confirm the suspected pathologies.

Point-of-care ultrasound (POCUS) of the abdomen performed by emergency physicians is increasingly being used to evaluate clinical manifestations and to facilitate accurate diagnoses in the emergency departments [3–6]. For children who present to the Pediatric Emergency Department (PED) with significant abdominal pain, a rapid distinction between emergencies and non-emergencies is vital to decrease morbidity and mortality. We describe a series of pediatric patients who presented to the PED with acute abdominal pain, in whom POCUS helped expedite the diagnosis by identifying varying types of intra-abdominal calcification and associated sonological findings. Using the case series for illustration, we hope to provide a general approach in the POCUS evaluation of intra-abdominal calcifications and their additional sonological findings in pediatric patients in the emergency department setting. For the narrative review of this article, we searched medical databases for studies of any design and in any setting that included pediatric patients describing POCUS, intra-abdominal calcification, and abdominal pain.

## Case presentation

### Case 1

A 10-year-old boy was brought to the PED with fever for four days with associated poor appetite and abdominal pain for three days. He had tenderness and guarding in the right iliac fossa. The patient was clinically suspected of having acute appendicitis.

**Fig. 1:**
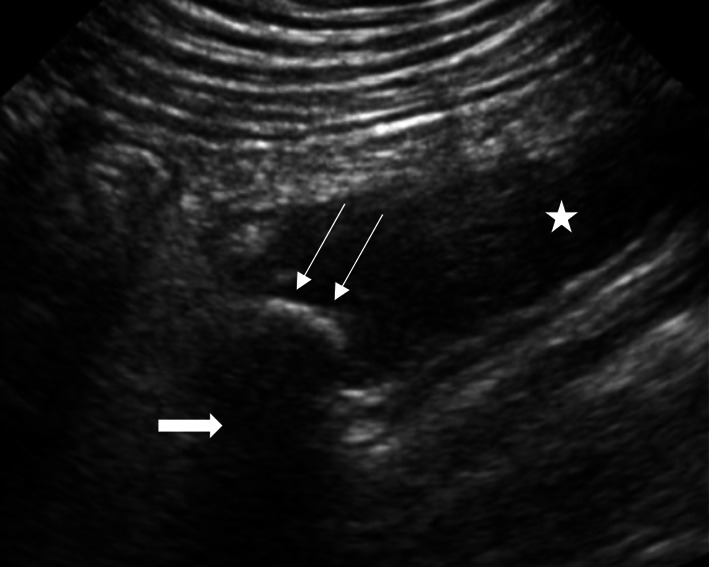
Appendicolith: Ultrasound abdomen of the right lower quadrant showing an appendicolith (long thin arrows), which is the echogenic area within the appendix lumen (distal appendix) (star). There is a posterior acoustic shadowing seen behind the echogenic foci (thick arrow). The largest appendix diameter measured was 16 mm

A POCUS examination of the abdomen was performed using a curvilinear transducer (2–5 MHz), which showed a distended appendix (16 mm in diameter) with wall thickening (Fig. 1) and associated probe-induced tenderness. An appendicolith with its posterior acoustic shadowing was seen in the distal appendix lumen (Fig. 1). A formal radiology ultrasound confirmed the diagnosis of acute appendicitis, and the patient was admitted for further inpatient management.

### Case 2

A 15-year-old adolescent girl was brought to the PED with fever for four days with progressively worsening right upper abdominal pain for three days. She had tenderness and guarding in the right hypochondria of the abdomen. She was clinically suspected of having acute cholecystitis.

**Fig. 2:**
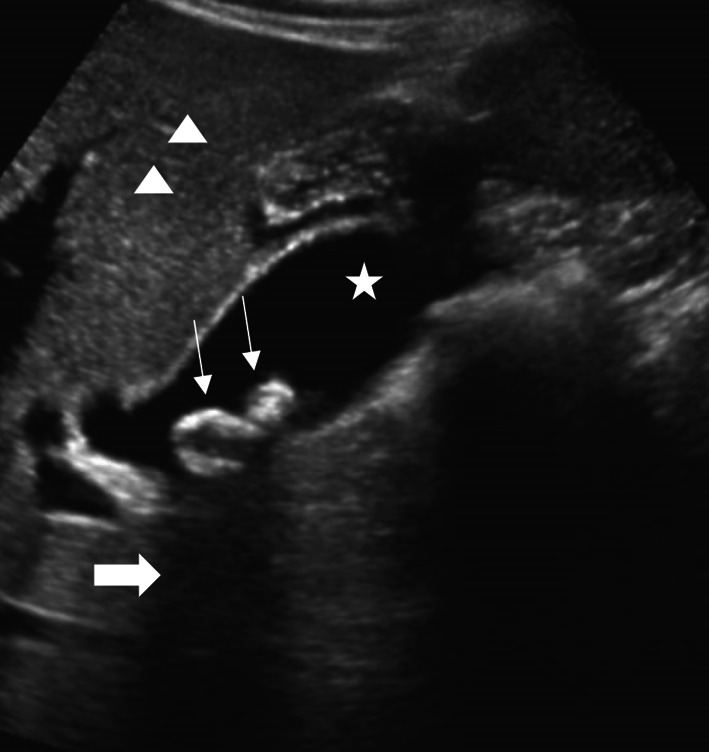
Gall bladder calculi: Gall bladder calculi appears as a highly reflective echogenic focus (long thin arrows) within gallbladder lumen (star), with prominent posterior acoustic shadowing (thick arrows). The liver parenchyma (arrowheads) can be seen on the superior aspect of the acoustic window

A POCUS examination of the abdomen was performed using a curvilinear transducer (2-5 MHz) and showed hyperechoic areas in the gall bladder with well-defined posterior acoustic shadowing (Fig. 2) and associated probe-induced tenderness. The patient was admitted for further management, and the radiological ultrasound confirmed acute cholecystitis.

### Case 3

A 13-year-old girl was brought to the PED with right upper abdominal pain for three days and fever for two days. She had similar abdominal pain in the past which resolved with oral analgesics. The examination of the abdomen revealed tenderness in the right hypochondria with guarding. She was clinically suspected of having acute cholecystitis.

**Fig. 3:**
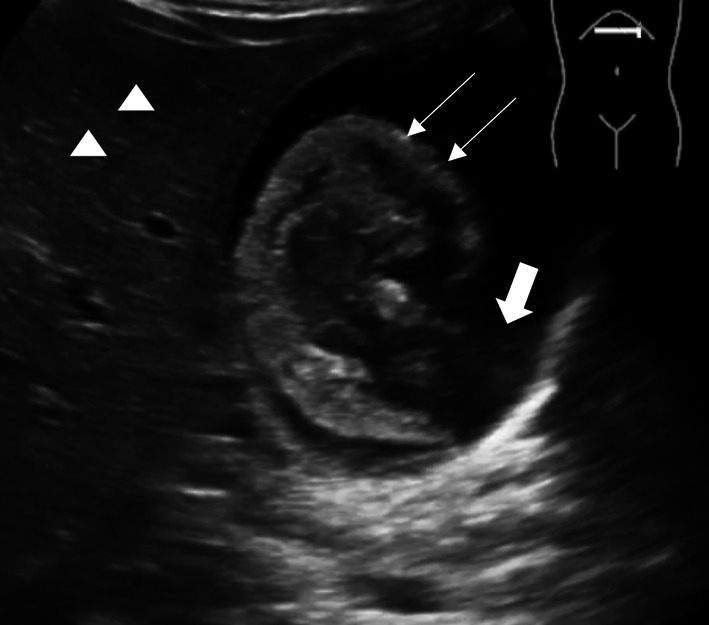
Gall bladder sludge: Gall bladder sludge (long thin arrows), which appears as low-amplitude homogeneous echoes, layering (thick arrow) on the posterior wall of the gall bladder with posterior acoustic enhancement due to change in reflective surface. The liver parenchyma (arrowheads) can be seen on the superior aspect of the acoustic window

A POCUS examination of the abdomen was performed using a curvilinear transducer (2–5 MHz), which showed hyperechoic areas in the gall bladder, which appeared as low-amplitude homogeneous echoes layering on the posterior wall of the gall bladder with no posterior acoustic shadowing suggestive of gall bladder sludge (Fig. 3) and associated probe-induced tenderness. The patient was admitted for further management, and formal radiological ultrasound confirmed the diagnosis of acute cholecystitis.

### Case 4

A 16-year-old boy presented to the PED with severe abdominal pain for the past six hours, radiating from the left lower abdomen to the groin area. Examination of the abdomen revealed tenderness in the left lumbar area with no guarding.

**Fig. 4:**
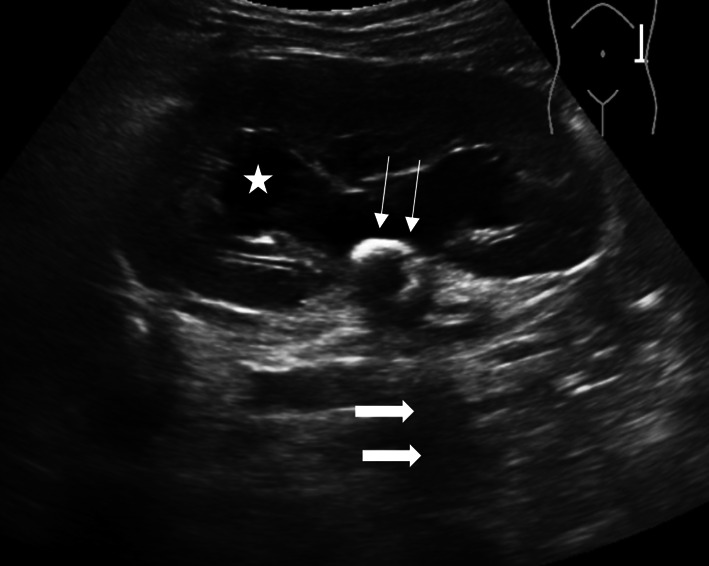
Renal calculi: Renal calculi seen in the renal pelvis as an echogenic focus (long thin arrows). There is associated posterior acoustic shadowing seen behind the echogenic foci (thick arrows). Pelvicalyceal dilation is also seen secondary to obstructive uropathy (star)

A POCUS examination of the abdomen performed using a curvilinear transducer (2–5 MHz), showed a hyperechoic area in the renal pelvis with pelvicalyceal dilatation (Fig. 4). A comprehensive ultrasound by the radiologist was suggestive of left-sided renal calculi with mild hydronephrosis.

### Case 5

A 2-year-old boy with underlying complex congenital cyanotic heart disease was brought into the PED with fever, abdominal pain, multiple episodes of non-bilious vomiting, and diarrhea for 3 days. His cardiac examination revealed a continuous murmur over the right upper sternal border. The room air oxygen saturation was 84%. The abdomen was distended and non-tender.

**Fig. 5:**
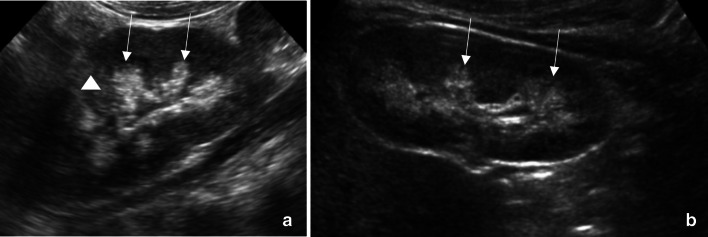
Nephrocalcinosis: **a** Right kidney: Ultrasound of the right kidney showing hyper-echogenicity of the renal pyramids with coarse echotexture (long thin arrows). There is no associated posterior acoustic shadowing seen. Loss of cortico-medullary differentiation (arrowheads) can also be seen. **b** Left kidney: Ultrasound of the left kidney showing hyper-echogenicity of the renal pyramids with coarse echotexture (long thin arrows)

A POCUS examination of the abdomen was performed using a curvilinear probe (2–5 MHz) to evaluate the abdominal distension and revealed no free fluid in the abdomen and no abnormally distended bowel loops. The renal POCUS showed calcification of the renal medulla in bilateral kidneys, appearing as hyperechoic areas in the renal pyramids with coarse echotexture, suggestive of suspected nephrocalcinosis (Fig. 5a, b). The patient was admitted for further workup, and the formal radiological ultrasound confirmed medullary nephrocalcinosis secondary to underlying medullary sponge kidney and current presentation due to viral gastroenteritis.

### Case 6

A sixteen-year-old girl presented to the PED with pain in the upper abdomen for three days. There was also associated severe nausea and three episodes of non-bilious vomiting. She had one episode of similar abdominal pain 2 years back and was managed conservatively. Her abdominal examination revealed tenderness in the epigastric region with guarding.

**Fig. 6:**
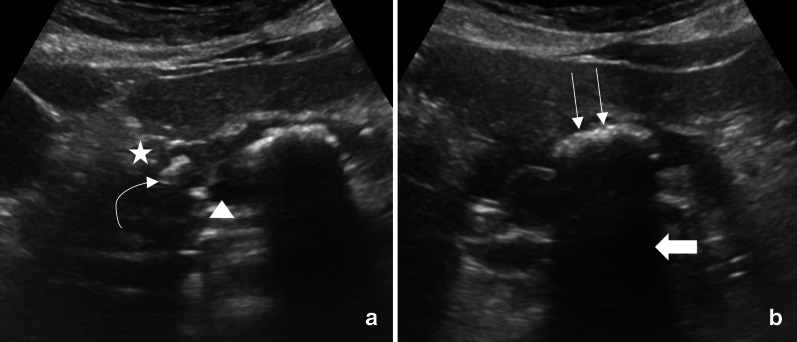
Calcific pancreatitis: **a** Heterogenous pancreatic tissue (star) with parenchymal calcifications (curved arrow) and cystic areas (arrowhead) seen in the pancreas. **b** Dense posterior acoustic shadowing (thick arrows) from intraductal calculus (long thin arrows) is also seen

An ultrasound examination of the abdomen was performed by an expert emergency physician trained in abdominal ultrasound, using a curvilinear probe (2–5 MHz), and showed a heterogenous pancreas with parenchymal calcification, cystic areas, and ductal calculus (Fig. 6a). Dense posterior shadowing from intraductal calculus was seen in the epigastric region (Fig. 6b). An initial impression of acute pancreatitis in the background of chronic calcific pancreatitis was considered. The patient was admitted for further workup, and formal radiology ultrasound confirmed the diagnosis of acute pancreatitis in chronic calcific pancreatitis.

## Discussion

POCUS examination of the abdomen performed by emergency physicians is increasingly being used to evaluate clinical manifestations and to facilitate accurate diagnoses in the emergency departments due to accessibility and portability of ultrasound machines [3–6]. The assessment of acute abdominal pain with POCUS must be done in conjunction with a thorough history and clinical examination generating a list of differentials as per the region of the abdomen. POCUS abdomen has been shown to aid and expedite the diagnosis in patients with abdominal trauma with hemoperitoneum, characterize systemic hypoperfusion and shock, detect gallstones, acute cholecystitis, and diagnose ureterolithiasis and assess for pyelonephritis [7–10]. It can be a useful adjunct for bedside diagnosis of digestive tract diseases such as intussusception, appendicitis, small bowel obstruction, and bowel perforation [3, 11–13]. POCUS can be a valuable imaging tool for assessing a pediatric patient with abdominal pain, in whom stringent adherence to the principle of ALARA is paramount [14]. A POCUS done by the emergency physician is shown to be associated with faster diagnosis time and expediting the disposition from the emergency department in a large variety of conditions [15, 16].

Abdominal POCUS is performed with the patient laying in the supine position. A low-frequency transducer is used to scan for focused findings (e.g., free fluid) [4]. A high-frequency linear probe can then be used to study the bowel, looking for abnormalities in the intramural areas and bowel wall thickening, as well as for the evaluation of the mesentery and surrounding lymph nodes [4]. The central abdominal areas are scanned to detect abnormal lymph nodes and abnormal dilatation of the small bowel [11]. After a thorough clinical history and physical examination, the differential diagnosis derived can guide further selective POCUS imaging of any of the following areas like the hepato-biliary structures, renal parenchyma, and bladder for hyperechoic lesions, calcifications, or abnormal dilation. The physician sonographer must bear in mind that these are not standard POCUS examinations. The presence of any abnormal sonological findings must be confirmed with a formal, comprehensive radiology ultrasound. This is especially important if the POCUS findings are out of the scope of Pediatric Emergency Medicine (PEM) POCUS, as currently described in the literature [4]. Additional scanning can be done in the subxiphoid window to evaluate for the presence of pericardial effusion and in bilateral upper quadrants for an anechoic area in the supradiaphragmatic area to detect the presence of pleural effusion [8].

POCUS is a valuable assessment tool for abdominal pain. The finding of different types of calcifications on POCUS can narrow down the differential diagnosis. When using POCUS to assess right lower quadrant pain, thoroughly interrogate for sonological features of acute appendicitis [4, 17]. The use of POCUS in these patients also demonstrated significantly decreased length of stay in the PED [4, 18]. The presence of a target sign with diameter > 6 mm, non-compressibility of the appendix, free fluid surrounding the appendix, and increased echogenicity of local mesenteric fat will facilitate the diagnosis of acute appendicitis [18, 19]. An appendicolith is a calcified deposit within the appendix, which may be an incidental finding, but it may also cause appendicitis [20]. On the ultrasound, an appendicolith will appear as a hyperechoic area within the appendix lumen, casting a posterior acoustic shadow (Fig. 1). Lack of consistent visualization of the appendix is a limitation when assessing the right lower quadrant, with the visualization rates ranging from 22 to 98% [21]. The use of additional sonological findings in these patients might help in the diagnosis.

POCUS of the hepato-biliary system is done to detect gallstones and evidence of biliary obstruction or inflammation [4]. Ultrasound is considered the gold standard for detecting gallstones [22]. On the greyscale ultrasound, the gallbladder calculi appear as a highly reflective hyperechoic focus within gallbladder lumen, with prominent posterior acoustic shadowing (Fig. 2) [23]. Upon changing the position of the patient, a gravity-dependent movement is often seen, and this is called the “rolling stone sign.” Gallbladder sludge appears as low-amplitude homogeneous echoes, layering on the posterior wall of the gall bladder (Fig. 3) [24]. The sludge does not cause posterior acoustic shadowing [25]. POCUS finding, which is most sensitive in acute cholecystitis, is the presence of cholelithiasis in combination with the sonographic Murphy sign, gallbladder wall thickening (> 3 mm), and pericholecystic fluid [4, 26]. A single large stone or a collection of stones within the gall bladder might result in “wall echo shadow” (WES sign), which results in failure to identify the gall bladder [4]. Biliary POCUS could be technically difficult to perform due to the presence of overlying fat, bowel gas, and abdominal discomfort [4]. The presence of gallstones might be an incidental finding on POCUS, and the PED physician must be vigilant not to overlook other life-threatening emergencies in children [4].

POCUS can be a useful adjunct in the assessment of the kidneys and urinary tract for the presence of hydronephrosis, calculi, cysts, and abscesses [4]. Urolithiasis is the presence of calculi anywhere along the course of the urinary tracts [27]. Ultrasound features of renal calculi include a hyperechoic focus in the renal pelvis (Fig. 4) or along the ureter or bladder. There is associated posterior acoustic shadowing seen behind the hyperechoic foci (Fig. 4). Ultrasound might not be able to detect the calcified stones in the ureters unless they are close to the renal pelvis or in close proximity to the bladder at the vesico-ureteric junction because the ureters are often covered by the bowel gas in the retroperitoneum [4, 28]. However, symptomatic patients with new-onset hydronephrosis and correlating abnormal bladder jet (either absent or very high/low velocity) might suggest a blocked ureter [28]. This is especially concerning if there are symptoms suggesting of urinary tract infection. Also, other differential diagnoses that must be considered in this situation are posterior urethral valves and external mass effect from constipated stool or tumor, which are beyond the scope of POCUS.

Nephrocalcinosis, which is the calcification of renal parenchyma, is seen as secondary to hyperparathyroidism, renal tubular acidosis, and medullary sponge kidney [29]. Ultrasound features of medullary nephrocalcinosis include hyper-echogenicity in the periphery of the pyramids in the early phase of the disease, progressing to coarse calcification in the late phase (Fig. 5) [30]. The presence of nephrocalcinosis is not a real PEM POCUS finding. The nephrocalcinosis can be seen in the PEM POCUS, but this is not included in the PEM POCUS syllabus [4]. A formal, comprehensive radiological ultrasound assessment must always confirm the incidental presence of renal parenchymal calcification detected on the POCUS examination.

Bedside ultrasound for assessment of the pancreas, and its pathology is out of the scope of PEM POCUS as currently described in the literature [4]. However, suggestive history and clinical findings, along with gross calcifications that might be seen in the upper abdomen, should raise the possibility of pancreatitis. Pancreatic calcifications are generally seen with chronic pancreatitis, although it can be seen in rarer conditions like hyperparathyroidism and hemochromatosis [31]. Typically, the calcification appears speckled (Fig. 6a) as the calcium gets deposited on the network of pancreatic ducts. The presences of intraductal calcifications with small cysts, which appear as hyperechoic foci in the epigastric region (Fig. 6b), combined with history and clinical features of recurrent pancreatitis, are pathognomonic for chronic pancreatitis.

In addition to the above causes of intra-abdominal calcifications, neuroblastoma, calcified lymph nodes, an ovarian dermoid, peri-portal calcified lymph node, fecalith, and calcified granulomas in the spleen will appear as a hyperechoic area on the POCUS imaging of the abdomen [1]. By correlating the location of the calcification with the underlying anatomical structure and integrating the findings with history and clinical details, pediatric emergency physicians can derive a focused set of differential diagnoses (Table 1).

**Table 1:** Differential diagnosis for calcified echogenic structures on POCUS abdomen in various anatomical areas

Right hypochondriac region	Epigastric region	Left hypochondriac region
Gall stones Peri-portal calcified lymph node Calcified granuloma liver	Chronic calcific pancreatitis (body)	Chronic calcific pancreatitis (tail) Calcified granuloma spleen

Right lumbar region	Umbilical region	Left lumbar region
Renal calculi Nephrocalcinosis Neuroblastoma	Calcified lymph nodes Neuroblastoma	Renal calculi Nephrocalcinosis Neuroblastoma

Right iliac region	Hypogastric region	Left iliac region
Calcified lymph nodes Appendicolith Ovarian dermoid Urinary tract calculi	Bladder calculi Vesico-ureteric junction calculi	Fecalith Calcified lymph nodes Ovarian dermoid Urinary tract calculi

There could be numerous pitfalls while performing POCUS abdomen in PED. The presence of abdominal pain might make the child uncooperative for the abdominal POCUS examination. Similarly, intestinal gas and fecal matter might obscure the underlying structures [32]. Calcifications on ultrasound can be challenging to detect or differentiate from bowel gas to the novice and even expert eyes. The posterior acoustic shadowing or signal void seen behind the calcified structures, which is an accessory finding in calcifications, will be absent in biliary sludge and nephrocalcinosis [23, 24, 29]. Misidentification of the anatomy might lead to misdiagnosis. For example, small bowel might be confused for appendix, portal vein or inferior vena cave or hepatic cysts can be mistaken for gall bladder, and ovarian cysts might be mistaken for urinary bladder [4].

## Conclusions

In a child presenting to the Pediatric Emergency Department with abdominal pain, POCUS and the findings of calcifications can narrow or expand the differential diagnosis when integrated with history and physical exam, to a specific anatomic structure. Integrating these findings with additional sonological findings of an underlying pathology might raise sufficient concerns in the emergency physicians to warrant further investigations for the patient in the form of a formal radiological ultrasound and assist in the patient's early disposition. The use of POCUS might also help to categorize the type of calcification to one of the four main categories of intra-abdominal calcifications, namely concretions, conduit wall calcification, cyst wall calcification, and solid mass-type calcification. POCUS used thoughtfully can give a diagnosis and expand differential diagnosis, reduce cognitive bias, and reduce physician mental load. By integrating the use of POCUS with the history and clinical findings, it will be possible to expedite the management in children who present to the Pediatric Emergency Department with acute abdominal pain.

## Data Availability

Data sharing not applicable to this article as no datasets were generated or analyzed during the current study.
